# Mechanism Analysis and Multi-Scale Protection Design of GaN HEMT Induced by High-Power Electromagnetic Pulse

**DOI:** 10.3390/mi13081288

**Published:** 2022-08-11

**Authors:** Lei Wang, Changchun Chai, Tianlong Zhao, Fuxing Li, Yingshuo Qin, Yintang Yang

**Affiliations:** Key Laboratory of Ministry of Education for Wide Band-Gap Semiconductor Materials and Devices, School of Microelectronics, Xidian University, Xi’an 710071, China

**Keywords:** GaN HEMT, damage effect, protection design, high-power electromagnetic pulse

## Abstract

Currently, severe electromagnetic circumstances pose a serious threat to electronic systems. In this paper, the damage effects of a high-power electromagnetic pulse (EMP) on the GaN high-electron-mobility transistor (HEMT) were investigated in detail. The mechanism is presented by analyzing the variation in the internal distribution of multiple physical quantities in the device. The results reveal that the device damage was dominated by different thermal accumulation effects such as self-heating, avalanche breakdown and hot carrier emission during the action of the high-power EMP. Furthermore, a multi-scale protection design for the GaN HEMT against high-power electromagnetic interference (EMI) is presented and verified by a simulation study. The device structure optimization results demonstrate that the symmetrical structure, with the same distance from the gate to drain (Lgd) and gate to source (Lgs), possesses a higher damage threshold compared to the asymmetrical structure, and that a proper passivation layer, which enhances the breakdown characteristics, can improve the anti-EMI capability. The circuit optimization results present the influences of external components on the damage progress. The findings show that the resistive components which are in series at the source and gate will strengthen the capability of the device to withstand high-power EMP damage. All of the above conclusions are important for device reliability design using gallium nitride materials, especially when the device operates under severe electromagnetic circumstances.

## 1. Introduction

The GaN high-electron-mobility transistor (HEMT) is a representative of wide-bandgap power semiconductor devices, which has great potential in high-frequency, high-power and high-temperature applications. This is because of the excellent properties of the GaN material [1], such as its higher electron mobility, saturation electron velocity and breakdown electric field, compared with Si and SiC [2,3,4,5,6,7,8]. The applications of GaN HEMT devices in harsh environments such as high-power microwave (HPM), high-power electromagnetic pulse (EMP) and particle irradiation make the reliability issues increasingly prominent.

Electromagnetic interference (EMI) is a typical reliability issue when an electronic system operates in a complex electromagnetic environment, which can easily access the system by the means of front-door (antenna) and back-door (microstrip line or power cable) coupling [9,10,11]. For a low-noise amplifier, the HEMT at the very front is the most vulnerable part when under an EMP injection [12,13]. Therefore, the study of EMI-induced damage effects on the GaN HEMT is of great significance.

In the past several years, a great deal of research projects have focused on damage effects induced by EMI on bipolar devices [14,15], CMOS inverters [16] and GaAs HEMTs [12,17], which have proposed a series of theoretical failure mechanisms and hardening designs. Kyechong K et al. [18] carried out a series of experimental studies of EMI effects and analyzed the mechanism on CMOS inverters induced by HPM. Chahine et al. [19] established a standard experimental device for measuring the interference threshold of IC with RF injection. Ma et al. [20] studied the damage mechanism and the relationship between energy and the pulse width of bipolar transistors under strong electromagnetic pulse injection. Yu et al. [21] analyzed the sensitivity to temperature and frequency induced by the latch effect of a CMOS inverter, as well as the failure mechanism of an AlGaAs/InGaAs HEMT under HPM injection. Qin et al. [22] studied the failure mechanism of enhanced and depleted AlGaN/GaN HEMTs under the action of HPM. Above all, it can be found that the damage effects and protection of GaN HEMTs under high-power EMP were rarely reported.

In this study, the underlying physical failure mechanism of the GaN HEMT under the injection of EMP is presented. Additionally, a series of protection studies were carried out with the help of the semiconductor simulation software TCAD (Sentaurus2013, Synopsys, California, US). First, we built a simulation model consisting of three parts: device structure, numerical model and circuit model. Following this, we conducted in-depth analysis on the failure mechanism of the GaN HEMT by extracting the variation in the internal electric field distribution, current density distribution and temperature distribution during the action of the high-power EMP. Finally, we put forward protective measures against the failure mechanism so as to improve the device reliability when operating under harsh environments.

## 2. Simulation Model

### 2.1. Structure Model

**Figure 1:**
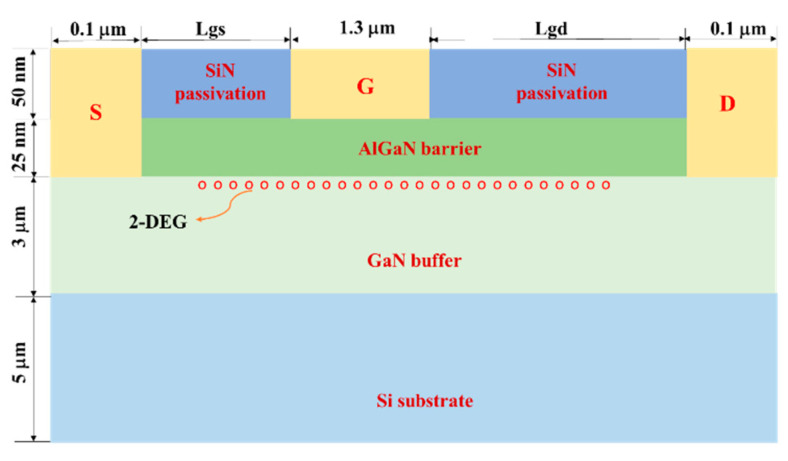
The geometric structure of the GaN HEMT.

Figure 1 shows the two-dimensional structure of the GaN HEMT studied in this paper, which consists of a 50 nm SiN passivation layer, a 25 nm AlGaN barrier layer, a 3 μm GaN buffer layer and a 5 μm Si substrate layer from top to bottom. The distance between the source and gate is referred to as Lgs, while that between the gate and drain is referred to as Lgd. The lengths of the drain, gate and source electrodes are 0.1 μm, 1.3 μm and 0.1 μm, respectively. The mole fraction x of Al_x_Ga_1-x_N in the proposed device is fixed at 0.2. The AlGaN barrier is uniformly doped with an N-type doping with a density of 1 × 10^17^ cm^−3^ impurities, forming a Schottky barrier with the gate metal. In order to form an ohmic contact, an N-type doping with a density of 1 × 10^20^ cm^−3^ is carried out under the drain electrode and source electrode. Bulk GaN exhibits slight N-type doping characteristics due to the formation of some oxygen or nitrogen vacancies during GaN epitaxial growth [23]. An N-type concentration of 2 × 10^16^ cm^−3^ is employed in the GaN buffer layer so as to make it equivalent to the actual situation. The thermal electrode is located at the bottom of the device in which the temperature is fixed at 300 K. The above two-dimensional model was verified in our previous work [22]. In this paper, the electrical characteristics of the device will no longer be discussed.

### 2.2. Numerical Model

With the help of TCAD, the burnout process of the GaN HEMT under a high-power EMP injection was simulated. The thermodynamic model (T-D) [24] dependent on temperature was activated to describe the carrier transport progress. In the T-D model, the Poisson equation, carrier continuity equations and heat flow equations were all solved in order to study the heating effect inside the device. In addition to Shockley–Read–Hall and Auger recombination dependent on temperature, mobility dependent on a high-field saturation model was also adopted [25,26]. Especially in AlGaN/GaN HEMTs, a high concentration of a two-dimensional electron gas already exists at the interface of the heterojunction in the absence of external stress, which is attributable to the spontaneous polarization and piezoelectric polarization [27]. The spontaneous polarization derives from the asymmetry of the hexagonal wurtzite structure of the GaN material, while the piezoelectric polarization derives from lattice mismatch during the growth of AlGaN on GaN [28]. The spontaneous and piezoelectric polarizations were taken into account using a built-in self-consistent polarization model [27] in TCAD. In the polarization model, the Poisson equation was modified by adding polarization charge to the right-hand side of the equation.

### 2.3. Circuit Model

**Figure 2:**
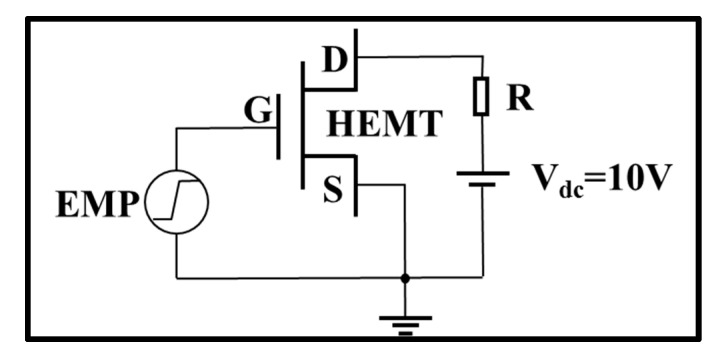
Schematic diagram of the circuit with the input injection.

The circuit model is shown in Figure 2. To simulate the damage effect of a GaN HEMT induced by a high-power EMP, a step voltage pulse was selected as the signal model, which has been proven to be equivalent to an EMP [29]. In this paper, the rising time and the amplitude of the step voltage pulse were set as 1 ns and 150 V to achieve high-power performance. The step voltage pulse was injected into the gate of the GaN HEMT. Meanwhile, the drain electrode was biased at 10 V, and the source electrode was grounded. The damage criterion was set as a lattice temperature of 1973 K during the simulation, which is in accordance with the melting point of the GaN material.

## 3. Results and Discussion

### 3.1. The Damage Effect and Mechanism Analysis

**Figure 3:**
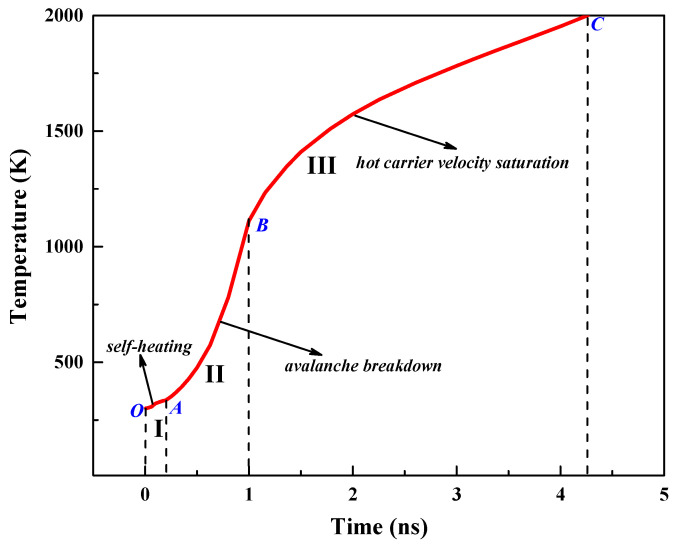
Variation in the maximum temperature (*T*_max_) inside the GaN HEMT with injection time.

To analyze the damage effects of the GaN HEMT due to the high-power EMP injection, the internal heating process of the device under the action of the EMP is discussed by taking the device structure when Lgs is 1 μm and Lgd is 3 μm. Figure 3 shows the temperature rise process inside the device. It can be clearly seen that the heating process of the device is divided into three stages, and the rate of temperature rise shows a “slow-sharp-fast” trend. At the beginning of the time, the temperature rises slowly, and this is defined as stage I (O–A segment); then, the temperature rises sharply, which is defined as stage II (A–B segment); in the last time period, the temperature rises fast, which is defined as stage III (B–C segment). This phenomenon can be explained by analyzing the variation in the internal distribution of multiple physical quantities in the device during the heating process.

**Figure 4:**
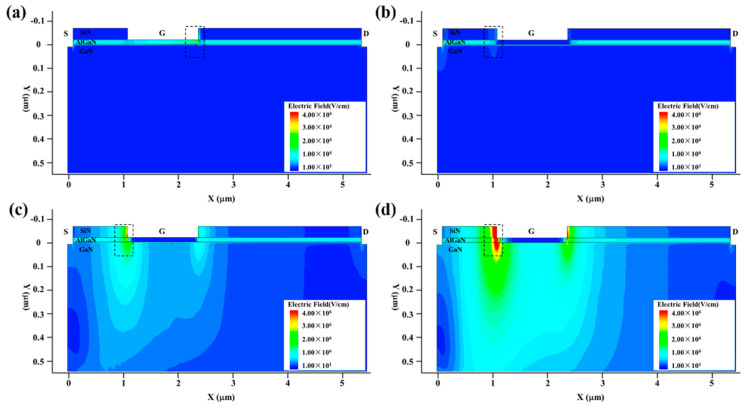
The internal electric field distribution of the device at various injection times: (**a**) 0 ns, (**b**) 0.1 ns, (**c**) 0.5 ns and (**d**) 2 ns.

**Figure 5:**
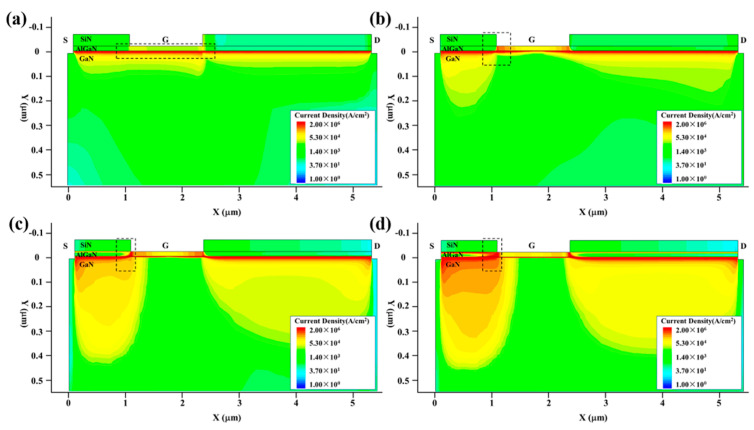
The internal current density distribution of the device at various injection times: (**a**) 0 ns, (**b**) 0.1 ns, (**c**) 0.5 ns and (**d**) 2 ns.

Figure 4 and Figure 5 show the internal electric field distribution and current density distribution of the GaN HEMT at the high-power injection times of 0 ns, 0.1 ns, 0.5 ns and 2 ns, which stand for the initial state, stage I, stage II and stage III. Before the EMP injection, the GaN HEMT was set at the work point of the source voltage (0 V), the gate voltage (0 V) and the drain voltage (10 V). This is the initial state of the GaN HEMT. As a depletion-type device, the channel is turned on, most of the carriers are concentrated in the two-dimensional electron gas (2DEG) layer and the voltage drop locates at the drain to gate and the drain to source. The electric field mainly distributes at the AlGaN layer and the corner from the gate to drain; the current density distribution mainly distributes at the channel layer, which is consistent with the results shown in Figure 4a and Figure 5a.

At the beginning of the step voltage pulse injection into the gate electrode, such as at the injection time of 0.1 ns, the Schottky junction is forward-biased and the electric field peaks are located at the left side of the gate, near the source, due to the fact that Lgd (3 μm) is larger than Lgs (1 μm), resulting in a current path appearing between the gate and the channel layer. The relevant results are displayed in Figure 4b and Figure 5b. As known, the Joule thermal power density P can be calculated by multiplying the electric field intensity E by the time current density J. Thus, the rise in temperature is determined by the electric field intensity and the current density distribution of the device. At the injection time of 0.1 ns in stage I, the electric field and the current are not much larger than the initial state; the rise in temperature occurs slowly, which may be attributed to the self-heating effect of the GaN HEMT.

With the increase in the injection time, once the increased injection voltage exceeds a certain value, the enhanced electric field strength will trigger avalanche breakdown and result in the current increasing rapidly. As shown in Figure 4c and Figure 5c, at the injection time of 0.5 ns in stage II, the electric field between the gate and source increases rapidly, and the current mainly flows to the source end through the two-dimensional electron gas channel at the AlGaN/GaN interface due to the electric field change. The enhanced electric field strength and current density resulting from the avalanche breakdown cause the temperature to rise sharply.

In stage III, with the increase in the pulse action time over 1 ns, the injection step voltage pulse reaches the voltage peak, and the electric field strength changes slightly. With the increase in the injection time, the thermal accumulation effect will cause the hot carrier emitter to appear and reach velocity saturation rapidly, due to the strong electric field. As shown in Figure 4d and Figure 5d, at the injection time of 2 ns in stage III, the slightly changed electric field strength, together with the velocity-saturated hot carrier, makes the temperature rise more slowly than in stage II. However, the temperature still rises very quickly due to the large electric field strength and current density.

**Figure 6:**
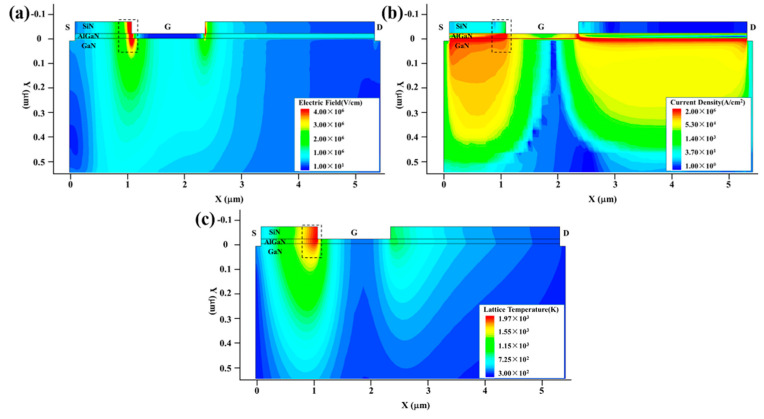
The internal (**a**) electric field intensity, (**b**) current density and (**c**) thermal distribution of the device at the moment of burnout.

Figure 6 shows the internal electric field intensity, current density and thermal distribution of the GaN HEMT at the moment of burnout. In Figure 6a,b, it can be seen that the maximum area of the electric field intensity is located at the gate corner near the source end, and that of the current density at the cylinder near the source end. These results reveal that the cylindrical surface of the gate corner near the source is the most vulnerable part due to the thermal accumulation effect which is consistent with the hot spot location of the device shown in Figure 6c. Similar results in a GaAs-based HEMT have been observed in our previous experimental study [12,17].

### 3.2. Multi-Scale Protection Design

According to the damage process analysis of the GaN HEMT by the high-power EMP injection, it can be found that the device damage is dominated by the different thermal accumulation effects during the action of the high-power EMP. Furthermore, the differences in the temperature rise process at various stages are associated with the different Joule thermal power densities P of the different thermal accumulation effects, where the thermal power density P is determined by the electric field intensity E and current density J. Thus, to achieve the protection design of the GaN HEMT against high-power EMP interference, the fundamental approach is to reduce the electric field intensity E and current density J inside the device so as to lower the thermal accumulation effect. Based on this principle, a series of multi-scale protection designs are proposed.

#### 3.2.1. The Device Structure Optimization Design

In order to regulate the electric field intensity E and current density J inside the GaN HEMT under the high-power EMP injection, one simple method is to change the size of the device. In this paper, we fixed the length of the drain, gate and source electrodes to 0.1 μm, 1.3 μm and 0.1 μm, respectively, and changed the source-to-gate distance Lgs and gate-to-drain distance Lgd to range from 1 μm to 3 μm in accordance with the total length of the device, which remained unchanged at 5.5 μm. Furthermore, five device structures were proposed, and their damage experiments were conducted in the TCAD simulation software. These device structures are (a) Lgs:Lgd = 3:1, (b) Lgs:Lgd = 2.5:1.5, (c) Lgs:Lgd = 2:2, (d) Lgs:Lgd = 1.5:2.5 and (e) Lgs:Lgd = 1:3.

**Figure 7:**
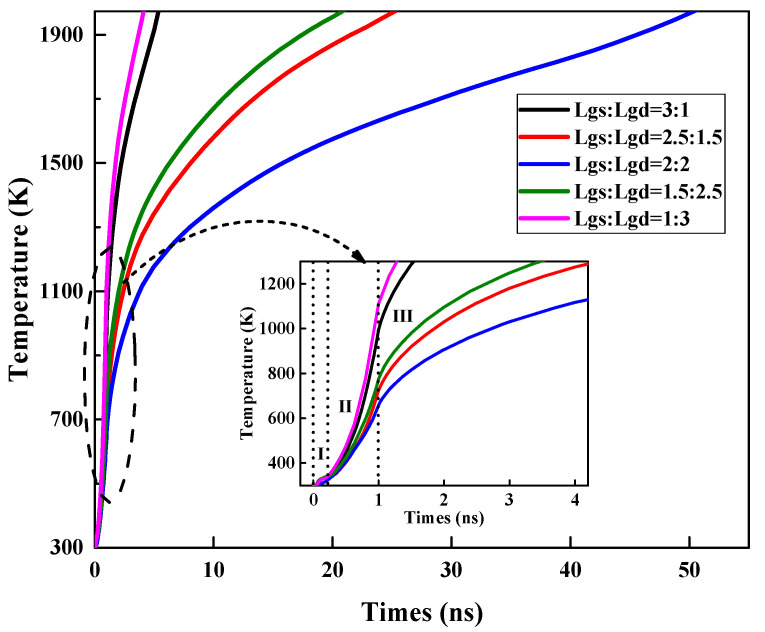
Variation in the maximum temperature (Tmax) inside the GaN HEMT with injection time for different device structures.

**Figure 8:**
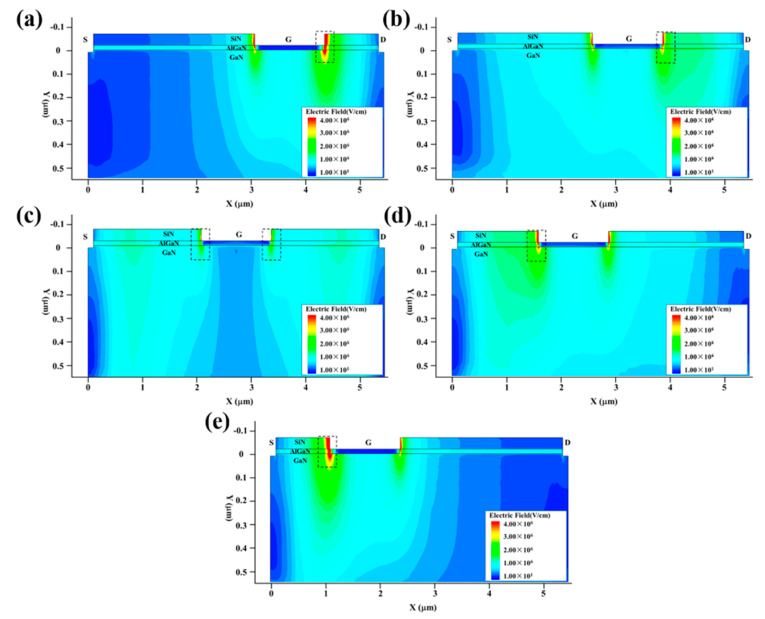
The electric field distribution of the GaN HEMT at the moment of burnout for different device structures: (**a**) Lgs:Lgd = 3:1; (**b**) Lgs:Lgd = 2.5:1.5; (**c**) Lgs:Lgd = 2:2; (**d**) Lgs:Lgd = 1.5:2.5; (**e**) Lgs:Lgd = 1:3.

**Figure 9:**
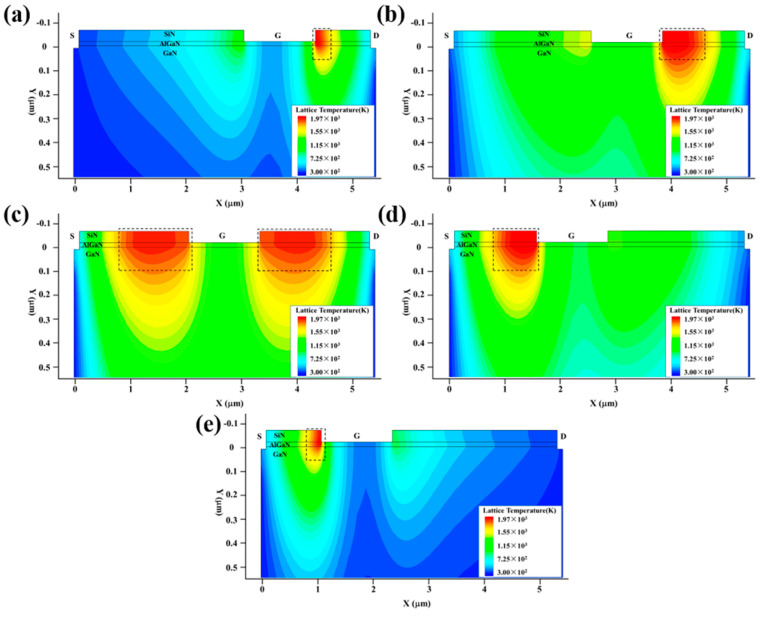
The thermal distribution of the GaN HEMT at the moment of burnout for different device structures: (**a**) Lgs:Lgd = 3:1; (**b**) Lgs:Lgd = 2.5:1.5; (**c**) Lgs:Lgd = 2:2; (**d**) Lgs:Lgd = 1.5:2.5; (**e**) Lgs:Lgd = 1:3.

**Figure 10:**
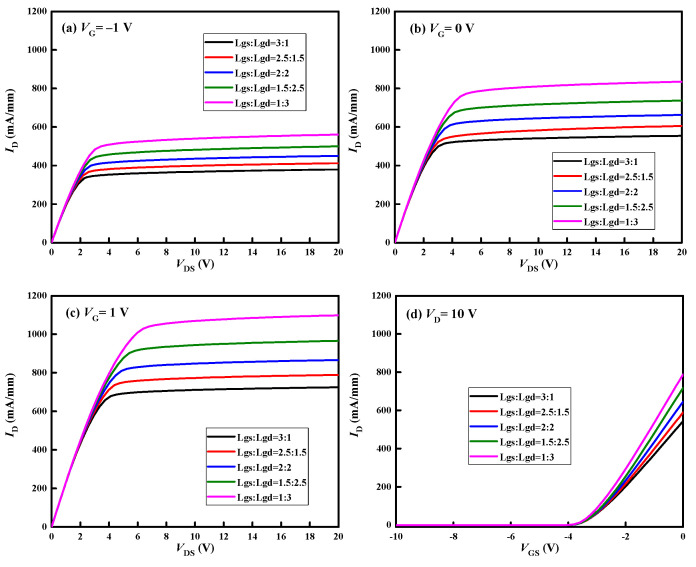
The output and transfer characteristics of the GaN HEMT for different device structures: (**a**) *I*_D_-*V*_DS_ at *V*_G_ = −1 V; (**b**) *I*_D_-*V*_DS_ at *V*_G_ = 0 V; (**c**) *I*_D_-*V*_DS_ at *V*_G_ = 1 V; (**d**) *I*_D_-*V*_GS_ at *V*_D_ = 10 V.

Figure 7 shows the variation in the maximum temperature (Tmax) inside the GaN HEMT with the injection time. Figure 8 and Figure 9 present the electric field and thermal distribution of the GaN HEMT at the moment of burnout for the above device structures. It can be seen that the heating processes of the different device structures are all divided into three stages, indicating the same damage mechanism, as discussed above. However, the burnout time, electric field distribution and thermal distribution change with the varied device structures. For structures (a) and (e), the burnout times are shorter than those of other structures, which can be attributed to them having the shortest Lgs or Lgd, resulting in a larger electric field intensity E than the other structures, as shown in Figure 8. Furthermore, the burnout time of structure (a) is longer than that of structure (e). This is because the voltage drop between the gate and source of structure (e) is larger than that between the gate and the drain of structure (a) due to the setting of the work point for the GaN HEMT during the high-power EMP injection, as discussed above. For structures (a) and (e), the maximum electric field strength is located on the left side of the gate near the drain and the right side of the gate near the source, respectively, which is consistent with the hot point distribution of the device shown in Figure 9. A similar phenomenon can also be found for structures (b) and (d). Furthermore, the symmetrical structure (c) with the same distance of the gate to drain (Lgd) and gate to source (Lgs) shows the longest burnout time, indicating a higher damage threshold than other asymmetrical structures. This is because the symmetrical structure (c) possesses a larger Lgd or Lgs compared to the asymmetrical structures, resulting in the minimum electric field strength under the same high-power EMP injection. In addition, the output and transfer characteristics of the GaN HEMT for the above five device structures were simulated, as shown in Figure 10. For the output characteristic *I*_D_-*V*_DS_ shown in Figure 10a–c, it can be seen that the saturated drain current (*I*_D_) changes with the different device structures, but the variation is only about 10% to 20% at a given gate voltage between the symmetrical structure and asymmetrical structure. Meanwhile, for the transfer characteristic *I*_D_-*V*_GS_ shown in Figure 10d, it can be seen that the threshold voltage of the GaN HEMT remains almost unchanged for the different device structures. These results can be attributed to the gates having the same length in the above device structures. Thus, the slightly changed output and transfer characteristics and the higher damage threshold of the device in a symmetrical structure make it a simple method of achieving the protection of the GaN HEMT against high-power EMP interference.

**Figure 11:**
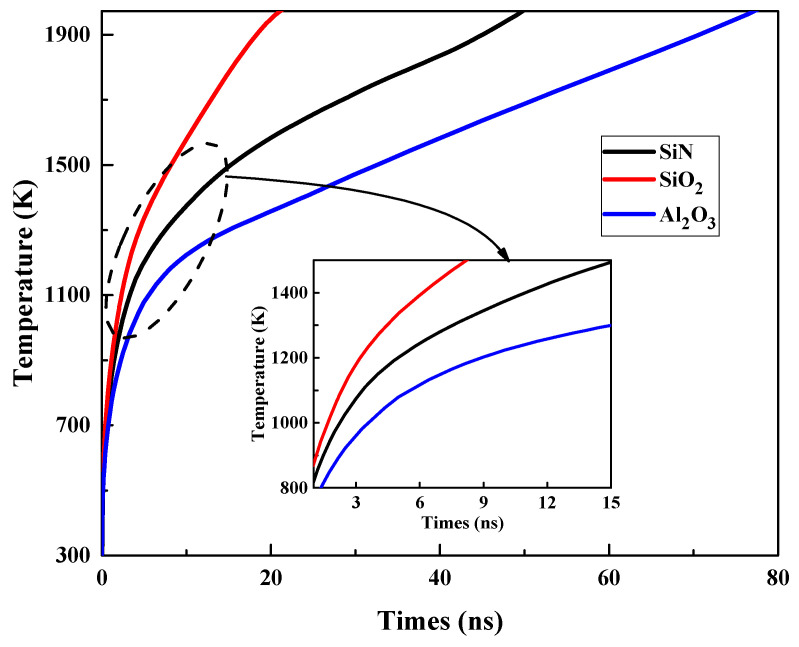
Variation in the maximum temperature (*T*_max_) inside the GaN HEMT with injection time for different passivation layers.

**Figure 12:**
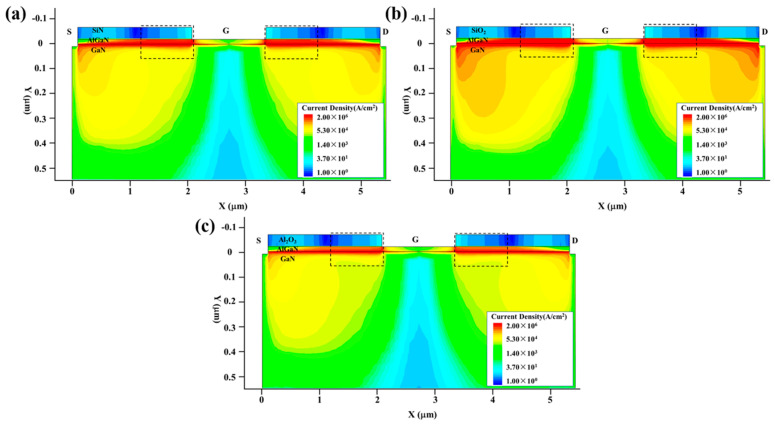
The current density distribution of the GaN HEMT at the moment of burnout with different passivation layers: (**a**) SiN; (**b**) SiO_2_; (**c**) Al_2_O_3_.

**Figure 13:**
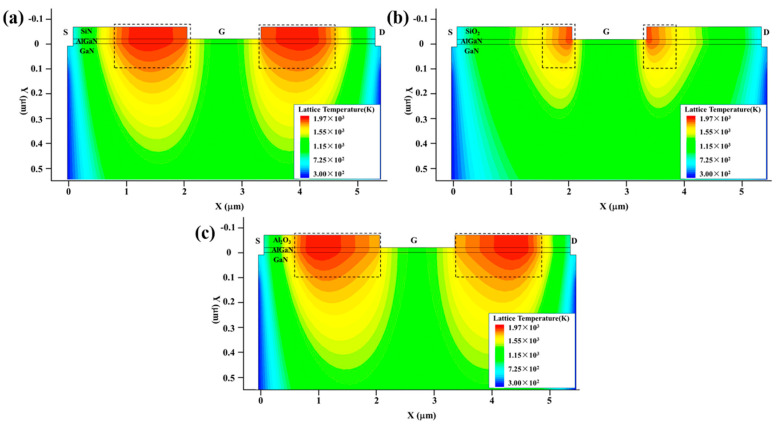
The thermal distribution of the GaN HEMT at the moment of burnout with different passivation layers: (**a**) SiN; (**b**) SiO_2_; (**c**) Al_2_O_3_.

To further enhance the anti-EMI capability of the symmetrically structured GaN HEMT, we can reduce the current density *J*, in addition to the reduction in the electric field intensity *E*. Based on the damage mechanism discussed above, we selected the symmetrical structure (c) and varied the passivation layer of SiN, SiO_2_ and Al_2_O_3_, which is compatible with the fabrication process, to reduce the gate–source and gate–drain currents which dominate the thermal accumulation in stage III during the damage process. A series of damage experiments were conducted in the TCAD simulation software. Figure 11 shows the variation in the maximum temperature (*T*_max_) inside the GaN HEMT with the injection time. Figure 12 and Figure 13 present the current density and thermal distribution of the GaN HEMT at the moment of burnout for the above device structures with different passivation layers. The similar temperature rise process and hot spot distribution demonstrate the same damage mechanism as discussed above. Furthermore, in Figure 11, it can be seen that the burnout times are about 20 ns, 50 ns and 80 ns for the devices with a passivation layer of SiO_2_, SiN and Al_2_O_3_, respectively, indicating the higher damage threshold of the device with the Al_2_O_3_ passivation layer than those with the SiO_2_ and SiN passivation layers. This can be explained as follows. As known, the thickness of the passivation layer and the permittivity of the passivation material dominate the breakdown performance of the GaN HEMT. In this work, the thickness of the passivation layer was fixed, whereas the permittivity *k* of the passivation material was varied for SiO_2_ at 3.9, SiN at 7 and Al_2_O_3_ at 9 [30]. The enhanced permittivity of the insulator will smoothen the electric field distributions along the barrier layer due to the uniform voltage drop across the high-*k* insulator [31]. Thus, the higher the *k* of the passivation material, the stronger the breakdown performance. That is to say, the devices with SiN or Al_2_O_3_ passivation layers possess improved breakdown performance, compared to that with a SiO_2_ passivation layer, due to the higher *k* of the passivation material. Based on the damage mechanism discussed before, the enhanced breakdown characteristics for the GaN HEMT will result in a reduction in the gate–source and gate–drain currents during the high-power EMP injection. These deductions are in accordance with the current density distribution shown in Figure 12. Therefore, the proper passivation layer choice can reduce the current density and the heat accumulation in the process of the high-power EMP injection, in turn improving the anti-EMI capability of the GaN HEMT.

#### 3.2.2. The External Circuit Optimization Design

**Figure 14:**
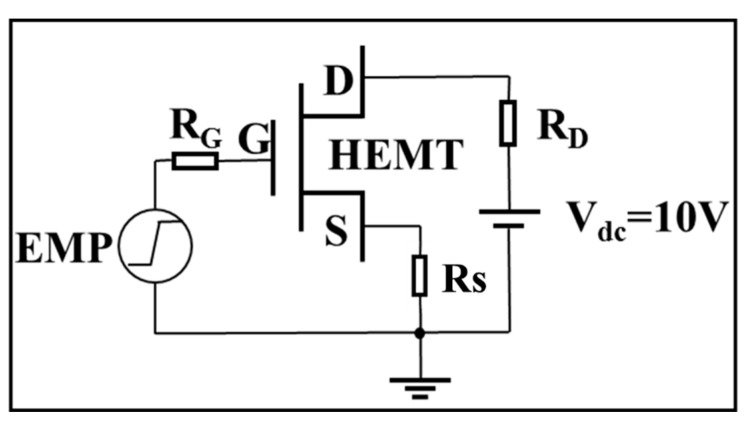
The simulation circuit with an external resistance R_G_ at the gate, R_D_ at the drain and R_S_ at the source.

In addition to the device structure optimization design, it is also possible to add some external components to the circuit to achieve the protection design of the GaN HEMT against high-power EMP interference. Figure 14 shows the simulation circuit with an external resistance R_G_ at the gate, R_D_ at the drain and R_S_ at the source. A series of damage experiments were conducted using the TCAD simulation software, with the following settings: R_G_ 1 kΩ, R_D_ and R_S_ 0.1 Ω, and vice versa.

**Figure 15:**
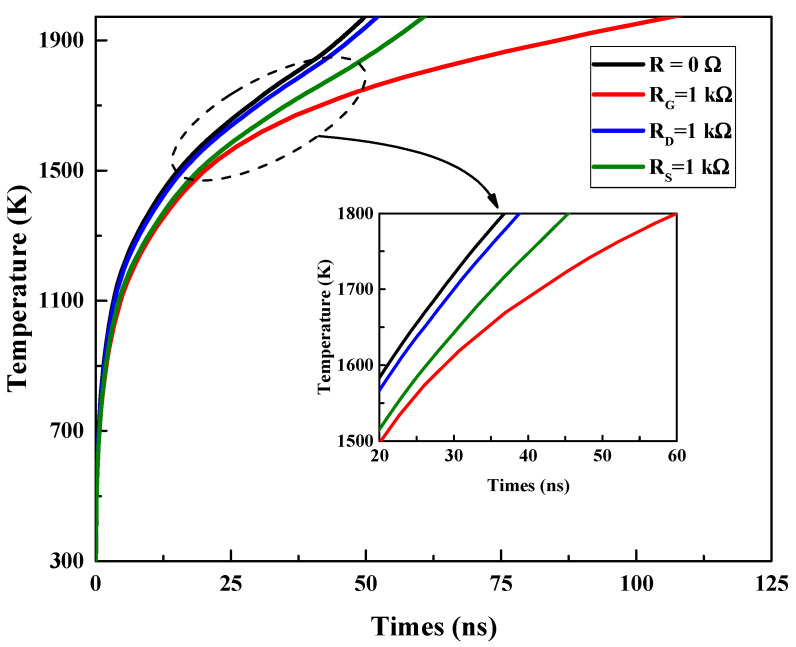
Variation in the maximum temperature (*T*_max_) inside the GaN HEMT with injection time in different external circuits.

**Figure 16:**
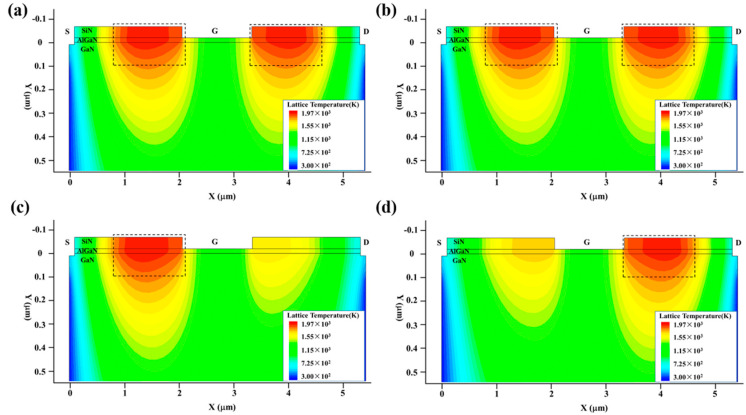
The thermal distribution of the GaN HEMT at the moment of burnout in different external circuits: (**a**) R = 0 Ω; (**b**) R_G_ = 1 kΩ; (**c**) R_D_ = 1 kΩ; (**d**) R_S_ = 1 kΩ.

Figure 15 shows the variation in the maximum temperature (*T*_max_) inside the GaN HEMT with the injection time, and Figure 16 presents the thermal distribution of the GaN HEMT at the moment of burnout for the symmetrical structure GaN HEMT with a SiN passivation layer in different external circuits. The similar temperature rise process and hot spot distribution demonstrate the same damage mechanism as discussed above. Furthermore, from Figure 15, it can be seen that the burnout time of the GaN HEMT under the same high-power EMP injection increases at varying degrees in different external circuits. This can be attributed to the reduction in the electric field intensity *E* and current density *J* inside the device when the external resistance is plugged in. Furthermore, the resistive component in series at the source exhibits a longer burnout time than that in series at the drain. This is because the GaN HEMT device is set at the work point of the source voltage (0 V), the gate voltage (0 V) and the drain voltage (10 V), before the high-power EMP injection, and the resistive component in series at the source will reduce the current density much more than that in series at the drain during the high-power EMP injection. In addition, the resistive component in series at the gate exhibits a longer burnout time than the others. This can be attributed to the direct thermal dissipation of the gate resistance during the high-power EMP injection into the device through the gate. Above all, these circuit optimization results illustrate that the resistive components which are in series at the source and gate will strengthen the capability of the device to withstand high-power EMP damage. Similar results in a GaAs-based HEMT have been observed in our previous study [29].

## 4. Conclusions

In this paper, a numerical simulation model was used to study the damage effect and failure mechanism of the GaN HEMT with a high-power EMP. The failure mechanism was presented by analyzing the variation in the internal distribution of multiple physical quantities in the device. The results reveal that the device damage was dominated by different thermal accumulation effects such as self-heating, avalanche breakdown and hot carrier emission during the action of the high-power EMP. As a result, to achieve the protection design of the GaN HEMT against high-power EMP interference, the fundamental approach is to reduce the electric field intensity E and current density J inside the device so as to lower the thermal accumulation effect. Based on this principle, a series of multi-scale protection designs were proposed and verified by a simulation study. The device structure optimization results demonstrate that the symmetrical structure possesses a higher damage threshold compared to the asymmetrical structure, and that the Al_2_O_3_ passivation layer, which enhances the breakdown characteristics, can improve the anti-EMI capability. The circuit optimization results demonstrate that the resistive components, which are in series at the source and gate, will strengthen the capability of the device to withstand high-power EMP damage. All of the above conclusions are important for device reliability design using gallium nitride materials, especially when the device operates under severe electromagnetic circumstances.

## Data Availability

The data used to support the findings of this study are available from the corresponding author upon request.
